# Chronic Conditions, Multimorbidity, and Health Outcomes Among US Adults

**DOI:** 10.5888/pcd22.250219

**Published:** 2025-07-03

**Authors:** Sara D. Turbow, Camille P. Vaughan

**Affiliations:** 1 Division of General Internal Medicine, Department of Medicine, Emory University School of Medicine, Atlanta, Georgia; 2 Division of Preventive Medicine, Department of Family and Preventive Medicine, Emory University School of Medicine, Atlanta, Georgia; 3 Division of Geriatrics & Gerontology, Department of Medicine, Emory University School of Medicine, Atlanta, Georgia; 4 Birmingham/Atlanta Geriatric Research Education and Clinical Center, US Department of Veterans Affairs, Atlanta, Georgia

Multimorbidity — living with multiple chronic conditions (MCCs) — is prevalent among adults in the US (1,2). In 2010, the US Department of Health and Human Services released a report, “Multiple Chronic Conditions: A Strategic Framework,” which presented an approach and future directions for the care and study of MCCs (3). Fifteen years later, we are still working to optimize that care.

This special collection of articles from *Preventing Chronic Disease* focuses on chronic conditions, multimorbidity, and health outcomes relevant to US adults. The collection includes 6 articles grouped around 2 themes: coordination of health care use and health disparities among patients with MCCs. The articles highlight several of the most pressing issues in multimorbidity research today, including the influence of including measurements of MCCs in studies related to prevention or treatment of other conditions, inclusion of people with MCCs in randomized controlled trials, and considerations for data sets available to understand MCCs in the US adult population. These articles discuss the need for continued advances in this area of research, practice, and policy, as well as some of the common challenges that arise when studying multimorbidity and chronic conditions.

## Health Care Use Among People With Multiple Chronic Conditions

Four articles in the collection focus on health care use among patients with MCCs. In “Health Care Use Among Cancer Patients with Diabetes, National Health and Nutrition Examination Survey, 2017–2020,” Jo et al use data from that survey to examine outpatient visits and hospitalizations among people with cancer, with cancer and prediabetes, or with cancer and diabetes (4). The results of the study were mixed, indicating that people with cancer and diabetes did not have more health care visits than those with cancer only. However, in adjusted models, people with cancer and prediabetes experienced 11% more health care visits than those with cancer alone, after controlling for demographics, body mass index (weight in kg divided by height in m^2^), having a usual source of care, education, financial status, health insurance status, chronic disease burden, and self-reported health status. The study points to the challenges and complexities of examining the interaction of 2 or more chronic conditions. The type of cancer, the treatments used, and the severity of diabetes were among the variables that could affect the relationship between diabetes, cancer, and health care use.

Next, in “The Joint Effect of Physical Multimorbidity and Mental Health Conditions Among Adults in Australia,” Ishida et al used data from the Household Income and Labour Dynamics in Australia Survey, a nationally representative, longitudinal survey focused on economic and personal well-being, labor market dynamics, and family life among people in Australia aged 15 years or older (5). They found that co-occurring multimorbidity and mental health conditions increased from 2009 to 2017 and that coexisting low socioeconomic status and multimorbidity and mental health conditions exacerbated the association between multimorbidity, health services use, and loss of work productivity and reduced health-related quality of life. That study showed that MCCs include both physical and mental health conditions and that a patient-centered approach that addresses management of both is likely needed to improve outcomes. This study also provides an important opportunity to examine MCCs in a global context and to consider how international studies of MCCs can be translated to a US context.

The last 2 articles that examine health care use and MCCs used data from the National Health Interview Survey. The first examines coordination of care among adults with epilepsy and other chronic conditions (6), and the second (7), investigates how a mental health diagnosis affects receipt of mental health care among adults with chronic kidney disease. In “Comorbidity Among Adults with Epilepsy — United States 2021–2022,” Zhou et al found that people with epilepsy were more likely to report 4 or more chronic conditions than those without epilepsy (6). In that study, the 1.8% of US adults with epilepsy had a higher prevalence of most chronic conditions except for diabetes, overweight, cancer, migraine, and skin allergy. Respondents with active epilepsy were more likely to report stroke, difficulty remembering, and migraine than those with inactive epilepsy and more likely to report all chronic conditions except overweight and current skin allergy compared with those with no epilepsy. Finally, in “Mental Health Symptoms and Receipt of Mental Health Care Among US Adults Diagnosed With Kidney Disease,” Villarroel et al used data from the 2021 National Health Interview Survey to compare the prevalence of depressive and anxiety disorders between respondents with self-reported kidney disease and those without kidney disease (7). In models adjusted for sociodemographic characteristics — including age, sex, ethnicity, educational attainment, marital status, family income, and urbanization level — adults with kidney disease were nearly twice as likely to have depression or anxiety than adults without kidney disease. However, when the models were also adjusted for health status and other chronic conditions — including coronary artery disease, diabetes, hyperlipidemia, and hypertension — the differences were no longer significant. Both articles highlight a core tension in research regarding MCCs: that the mix of chronic diseases matters more than the number of chronic diseases a person has and that studies should seek to identify and analyze clusters of MCCs rather than just their total number.

## Health Disparities in Patients with MCCs

The last 2 articles in the collection focus on health disparities among adults with MCCs. First, in “Racial and Ethnic Disparities in Use of Colorectal Screening Among Adults with Chronic Medical Conditions: BRFSS 2012–2020,” Castañeda-Avila et al use the Behavioral Risk Factor Surveillance System to analyze differences in the use of colorectal cancer screening based on the presence of 1, 2, 3, or 4 or more chronic conditions. The study included Hispanic adults with low English proficiency, Hispanic adults proficient in English, White adults, and Black adults (8). Notably, 66.5% of respondents were up to date with screening. Chronic conditions were common, with approximately 15% of all respondents having 4 or more. Overall, the prevalence of being up to date increased as the number of chronic conditions increased. Overall, Hispanic respondents with low English proficiency had the lowest screening prevalence (15% lower than the comparison group of non-Hispanic White patients). The study highlights a challenge in examining multimorbidity — that patients with MCCs must have access to and engagement with the health care system. As observed in this study, chronic conditions that are associated with more frequent health care encounters may lead to better adherence to preventive care recommendations. However, social determinants of health remain a major factor, as observed in the low English-proficiency group, which had the lowest screening prevalence.

Finally, in “Social Deprivation and Multimorbidity Among Community-Based Health Center Patients in the United States,” Valenzuela et al (9) used electronic health record data from 678 community-based health centers in 27 states, recorded from 2012 through 2019, to assess the relationship between geography and number of MCCs in adults aged 45 years or older. Overall, no underlying pattern was observed in the geographic distribution of multimorbidity measured as a count of conditions. However, compared with non-Hispanic White patients, the study observed higher multimorbidity in socially deprived geographic areas, and after controlling for patient- and area-level sociodemographic indicators, electronic health records showed lower numbers of chronic diseases among non-Hispanic Black patients, Hispanic patients conversant in both English and Spanish, and non-Hispanic Asian patients. The study highlights the importance of using granular data, ideally at the individual level, to understand the influence of factors contributing to health disparities, including both sociodemographic indicators and geographic areas that have a higher prevalence of MCCs.

The studies in this collection highlight several critical aspects of current MCC research. First, not all MCCs are created equal, and studies need to account for disease burden in their population selection and study design. The included studies that looked at MCC burden by number of conditions (Valenzuela [9], Zhou [6], and Castañeda-Avila [8]) were more likely to observe a mix of positive and negative results. We encourage researchers to consider common coexisting clusters of MCCs (10) in their analyses to reflect patterns seen at the population level and to increase the effectiveness of their work. Second, the ability to assess the influence of social determinants of health (SDOH) provides critical information to understand potentially reversible factors influencing gaps in health outcomes among people with MCCs. A challenge, however, is that SDOH data are not always available at the patient level. Several studies in this special issue used national survey data — data sets often supported by the Centers for Disease Control and Prevention — to not only obtain national perspectives on MCCs, but also assess individual-level SDOH. These data sets are critical to this work. To continue to move the field of MCC research forward, we must continue to advance our approaches, including pursuing prospective studies, advocating for the inclusion of patients with MCCs in randomized controlled trials, and maintaining and expanding our national data infrastructure to improve health for all.

## References

[1] Wolff JL , Starfield B , Anderson G . Prevalence, expenditures, and complications of multiple chronic conditions in the elderly. *Arch Intern Med.* 2002;162(20):2269–2276. 10.1001/archinte.162.20.2269 12418941

[2] Maciejewski ML , Hammill BG . Measuring the burden of multimorbidity among Medicare beneficiaries via condition counts and cumulative duration. *Health Serv Res.* 2019;54(2):484–491. 10.1111/1475-6773.13124 30790281 PMC6407342

[3] US Department of Health and Human Services. Multiple Chronic Conditions: a Strategic Framework. 2010. Accessed May 21, 2025. https://www.hhs.gov/sites/default/files/ash/initiatives/mcc/mcc_framework.pdf.

[4] Jo A , Parikh S , Sawczuk N , Turner K , Hong YR . Health care use among cancer patients with diabetes, National Health and Nutrition Examination Survey, 2017–2020. *Prev Chronic Dis.* 2024;21:E58. 10.5888/pcd21.240066 39117352 PMC11318949

[5] Ishida M , Hulse ESG , Mahar RK , Gunn J , Atun R , McPake B , . The joint effect of physical multimorbidity and mental health conditions among adults in Australia. *Prev Chronic Dis.* 2020;17:E157. 10.5888/pcd17.200155 33301391 PMC7769083

[6] Zhou Y , Kobau R , Pastula DM , Greenlund KJ . Comorbidity among adults with epilepsy — United States, 2021–2022 . *Prev Chronic Dis.* 2024;21:E100. 10.5888/pcd21.240313 39700074 PMC11675796

[7] Villarroel MA , Wang X . Mental health symptoms and receipt of mental health care among US adults diagnosed with kidney disease. *Prev Chronic Dis.* 2025;22:240509. 10.5888/pcd22.240509 PMC12249301 40609021

[8] Castañeda-Avila MA , Tisminetzky M , Oyinbo AG , Lapane K . Racial and ethnic disparities in use of colorectal cancer screening among adults with chronic medical conditions: BRFSS 2012–2020. *Prev Chronic Dis.* 2024;21:E12. 10.5888/pcd21.230257 38386629 PMC10890357

[9] Valenzuela S , Peak KD , Huguet N , Marino M , Schmidt TD , Voss R , . Social deprivation and multimorbidity among community-based health center patients in the United States. *Prev Chronic Dis.* 2024;21:E75. 10.5888/pcd21.240060 39325637 PMC11451564

[10] Weiss CO , Boyd CM , Yu Q , Wolff JL , Leff B . Patterns of prevalent major chronic disease among older adults in the United States. *JAMA.* 2007;298(10):1160–1162. 10.1001/jama.298.10.1160-b 17848649

